# Dietary supplementation of black rice bran to colon carcinogen-induced mice: Examining the development of colorectal cancer by improving environmental colon conditions

**DOI:** 10.1016/j.heliyon.2023.e18528

**Published:** 2023-07-28

**Authors:** Slamet Budijanto, Winda Nurtiana, Amirotul Muniroh, Yeni Kurniati, Lilis Nuraida, Bambang Pontjo Priosoeryanto, Fitriya Nur Annisa Dewi, Ardiansyah Ardiansyah, Nancy Dewi Yuliana, Safrida Safrida, Hitoshi Shirakawa

**Affiliations:** a Department of Food Science and Technology, IPB University, Dramaga, Bogor, 16680, Indonesia; b Department of Food Technology, University of Sultan Ageng Tirtayasa, Raya Jakarta Km 4 Pakupatan, Serang, 42124, Indonesia; c Department of Food Technology, University of PGRI Wiranegara, Ki Hajar Dewantara, Pasuruan, 67118, Indonesia; d Department of Food Science and Technology, Indonesian Tourism Academy, Duren Sawit, Jakarta, 13620, Indonesia; e Southeast Asian Food and Agricultural Science and Technology (SEAFAST) Center, IPB University, Dramaga, Bogor, 16680, Indonesia; f Department of Veterinary Clinics, Reproduction, and Pathology (CRP), IPB University, Dramaga, Bogor, 16680, Indonesia; g Primate Research Center, IPB University, Lodaya II/5, Bogor, 16151, Indonesia; h Department of Food Science and Technology, Bakrie University, Kawasan Epicentrum, Jakarta, 12920, Indonesia; i Department of Public Health, University of Teuku Umar, Meureubo, Meulaboh, 23681, Indonesia; j Laboratory of Nutrition, Graduate School of Agricultural Science, Tohoku University, Aoba-ku, Sendai, 980-0845, Japan; k International Education and Research Center for Food Agricultural Immunology, Graduate School of Agricultural Science, Tohoku University, Aoba-ku Sendai, 980-0845, Japan

**Keywords:** Black rice bran, β-Glucuronidase, Colorectal cancer, Short-chain fatty acids, Histopathology, Immunohistochemistry

## Abstract

This research aims to identify the effects of the administration of a black rice bran diet on colorectal cancer in dextran sodium sulfate and azoxymethane-induced BALB/c mice. The research was conducted on three groups consisting of eight Balb/c mice: two groups were fed with carcinogens, and the third group, referred to as the normal group, was supplied with Isotonic NaCl 0.9% intraperitoneally. One group fed with carcinogens was supplied a standard AIN 1993 M diet modified with black rice bran as a substitute of fibre source, while the other two mice groups were fed the standard diet (AIN-93M) containing cellulose fibre. At the 17th week, all mice were euthanized; their colonic sections were taken for histopathological evaluation, and cecum for short-chain fatty acids concentration, total lactic acid bacteria, pH and β-glucuronidase activity evaluations. The results show an increase in the total lactic acid bacteria and short-chain fatty acids in the mice group fed with rice bran. Consequently, pH value and β-glucuronidase activity had decreased. Histopathological evaluation of mucosal tissue exhibited inhibition of the tumor growth rate in the mice groups fed rice bran compared to the group supplied with the standard diet. Furthermore, the proliferating cell nuclear antigen expression had decreased significantly, while expression of caspase-8 and caspase-3 had increased notably, in the group fed with a rice bran diet. These results suggest that black rice bran can effectively inhibit colon carcinogenesis. The potential of black rice bran as a source of fibre has not been studied in detail regarding the inhibition mechanism of colorectal cancer cells; further investigation in this field could provide valuable information about new strategies to prevent colorectal cancer. This strand of research is very important to developing preventive methods against cancer and promoting the concept of healthy products, including functional foods.

## 1 Introduction

Cancer is estimated to be the leading cause of death out of all non-communicable diseases. Colorectal cancer is ranked the second most common cause of death, and third of incidence, with the highest occurrence of colon cancer cases in Europe, followed by Australia, America and Asia [1]. Previous studies suggest that the consumption of wholegrains or food containing dietary fibres is negatively correlated with the risk of colon and rectal cancer [2].

Dietary fibre and bioactive compounds such as polyphenols, flavones (quercetin, kaempferol, apigenin), isoflavones, caffeic acid, and resveratrol are known to act as chemopreventive agents preventing colorectal cancer both in vitro and in vivo, respectively [3]. Dietary fibre can bind carcinogen of secondary bile acid, increasing the faecal bulk (diluting the carcinogenic agent), and preventing carcinogens from coming into direct contact with the colon for an extended period of time [4]. Bioactive compounds are widely known to reduce several chronic diseases and enhance the antiproliferation of colon cancer [5]. In some recent scientific studies, whole-grains have been considered as healthy foods because of their phytochemical and dietary fibre content [6]; one particular grain known for its health benefits is the rice grain [7].

Phytochemical compounds in rice accumulate in aleurone (bran), and bran is known to contain various bioactive compounds, such as flavonoid compounds, phytic acid, γ-oryzanol, vitamin E complex, vitamin B complex and phytosterol [8]. Rice bran also contains dietary fibre with approximately 21.2–30.2% (21.17% insoluble and 2.17% soluble) [9,10].

Several studies show that black rice bran has substantially greater antioxidant activity from anthocyanin pigments in the aleurone layer, which gives it its violet or dark purple color [11,12]. Compared to other colored rice (white, red, and brown rice), black rice has higher fiber content that could bind bile acids and carcinogens and repair the mucosal epithelium of the colon [13,14]. The dietary fiber not only could reduce stool transit time but also increase fermentation which produces short-chain fatty acids (SCFAs) by colonic microbiota, which stimulates repair and maintains the colon wall’s health [13,15].

In this study, we evaluate the potential of black rice bran in the prevention of colon cancer. In our previous studies, black rice bran exhibited antiproliferation activity in WiDr colon cancer cells [16,17]; therefore, this continuation of our studies aims to determine the effect of supplying the black rice bran diet as a source of fibre in inhibiting the development of colon cancer in BALB/c mice, compared to those fed with a cellulose fibre diet.

## 2 Materials and methods

### 2.1 Preparation of black rice bran sample

The specific black rice bran chosen was of the ‘Cempo Ireng’ variety from farmers in Cigudeg, Bogor, Indonesia. It is one of the local pigmented rice varieties which has a high content of total flavonoids, anthocyanins, and phenolics [18,19]. The grain was milled by a huller machine (HW-60A, Yanmar Diesel Engine, Co., LTD, Osaka, Japan) and polished for 2 min by a rice polisher (N-70F, Satake Engineering Co., LTD, Tokyo, Hiroshima). Next, the bran was dried with a freeze dryer (Labconco, Labconco Corporation, Kansas, USA) for 24 h, and stored in a freezer at −18 °C until it was ready to be mixed into a diet. Black rice bran’s total dietary fiber content is 23.06% (6.89% soluble dietary fiber and 16.17% insoluble dietary fiber).

### 2.2 Animals

All procedures involving the care and use of laboratory animals were performed following the approval from Bimana Indomedical Institutional Animal Care and Use Committee, Bogor, Indonesia (approval number R.02-17-IR). Twenty-four male BALB/c mice (5–8 weeks old) were obtained from the Indoani Lab (Bogor, Indonesia), classified into three groups (n = 8 per group), placed individually in rooms regulated by air and light cycles. During acclimation in a week, all mice were fed a standard diet. After that, only the BRB group were further adapted to the modified diet with a fibre source of black rice bran. Following the third week (pre-induction), carcinogen-induced group (C+) and BRB mice were injected intraperitoneally with azoxymethane (AOM) (Sigma Aldrich, Merck, St. Louis, MO, USA) at a dose of 10 mg/kg body weight.

The standard diet based on the AIN-93 M standard, rations should contain 100% of the following: 14% protein, 4% fat, 5% food fiber, 3.5% minerals, 1% vitamins, 10% sucrose, and carbohydrates [20]. The findings of proximate analysis of sodium caseinate and black rice bran, which include water, lipid, protein, ash, dietary fiber, and carbohydrate content by difference, were used to calculate the rations case.

**Fig. 1:**
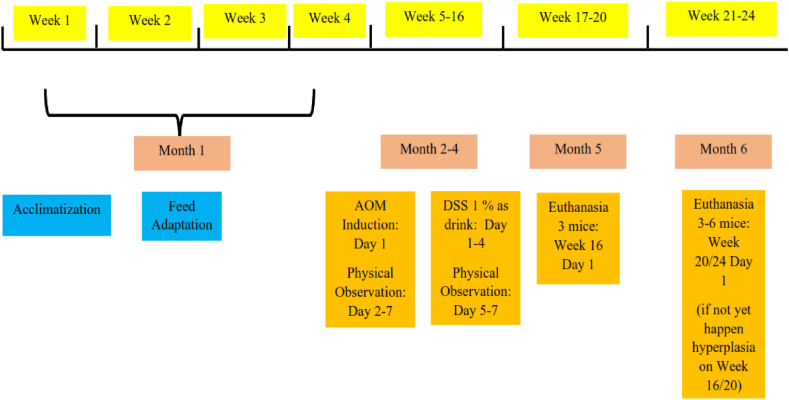
Cancer formation protocol.

The normal (C-) group was given Isotonic NaCl 0.9% (Ecosol NaCl, Braun Pharmaceutical, Karawang, Indonesia) intraperitoneally to settle their stress levels. One week after induction, the C+ and BRB groups were given 1% Dextran Sulfate Sodium (DSS) (MP-Bio, Solon, Ohio, USA) in their drinking water by *ad libitum* for four consecutive days to accelerate the occurrence of colon cancer. The cancer formation protocol can be seen in Fig. 1. The diet was supplied according to the daily nutrition of mice, which was 15 g/100 g of body weight with diet composition, as shown in Table 1.

**Table 1:** Diet composition (referred to the AIN-93 M standard composition).

Ingredients (g/100 g)	Standard diet	Modified diet
Black rice bran	0.00	21.68
Sodium caseinate	14.00	11.48
Cellulose powder	5.00	0.00
Soybean oil	3.98	1.67
Vitamin mix	1.00	1.00
Mineral mix	2.99	1.81
Cornstarch	63.03	52.35
Sucrose	10.00	10.00

Normal group (C−) and carcinogen-induced group (C+) given cellulose as fibre source, BRB group given black rice bran as fibre source.

During the study, the mice were observed intensively in terms of their physical condition, body weight, and health, including their activity, appetite and the condition of faeces. Mice faeces collected from the bedding in the span of three days were used to evaluate the SCFAs concentration and pH values, while faeces obtained directly from the anus were used to evaluate the total of lactic acid bacteria (LAB).

At the end of this study, after being induced by AOM at the 17th week, all mice were euthanized; their colons were taken and analyzed for the histopathology of colon cancer, and the cecum contents were analyzed to examine the SCFAs concentration, the total of LAB and pH values. Next, parts of the cecum walls were analyzed to investigate β-glucuronidase activity. All treatment procedures were in accordance with the standard operating rules and approved by the Welfare Supervision Commission and Use of Research Animals of PT Bimana Indomedical, Bogor, Indonesia (R.02-17-IR.).

### 2.3 Determination of the total of LAB

Determining the total of LAB was conducted following the example of Aattouri et al. [21]. The sample of cecum contents or faeces which had been diluted were poured deMan- Rogosa-Sharpe agar steril (MRS; Oxoid, Basingstoke, UK), previously diluted with CaCO3 1% (Merck, Darmstadt, Germany), and then incubated at 37 °C for 48 h. LAB had a clear zone around it. The addition of CaCO3 aims to indicate the growth of LAB by forming a clear zone around the colony. LAB were counted by a single colony and converted to Log CFU/g.

### 2.4 Determination of pH

An amount of 0.7 g of cecum contents or faeces were homogenized with 5 mL of distilled water using vortex (VM-10, WiseMix, Witeg Labortechnik, GmbH, Wertheim, Germany) for 10 min; this solution was then measured using a pH meter (Ohaus ST3100MF, Ohaus Corporation, New Jersey, USA).

### 2.5 Determination of SCFAs concentration

The determination of (SCFAs) concentration was conducted following the procedure of Filipek and Dvorak [22]. A sample of 0.7 g of cecum contents or faeces was homogenized with 5 mL distilled water for 10 min; next, the solution of H_2_SO_4_ (Merck, Darmstadt, Germany) and 0.003 g of sulfuric acid-5-salicylate dihydrate (Sigma Aldrich, Merck, St. Louis, MO, USA) were added to the 1 mL sample (up to pH 3–4). The solution was centrifuged (Sorvall Legend micro 17R high speed refrigerated centrifuge, Thermo Fisher Scientific, Waltham, Massachusetts, USA) for 10 min at the speed of 16,480×*g* at 7 °C, and 0.6 mL of supernatant was injected into gas chromatography with flame-ionization detector (Chrompack CP 9002 seri 946253, Chrompack, Middelburg, Netherlands). GC condition using a flame ionization detector and a capillary column. Nitrogen was used as the carrier gas at a 30 mL/min flow rate. The initial temperature of the column was 60 °C, then increased by 20 °C per min until up to 200 °C. The injector temperature is 250 °C and the detector temperature is 270 °C. The sample injection time is 9 min. Standard (SCFAs) contain a mixture of acetic, propionic, and butyric.

### 2.6 Determination of β-glucuronidase activity

The determination of β-glucuronidase activity was conducted following the procedure of Jenab and Thompson [23]. A sample of 0.5 g of cecum wall was homogenized with 10 mL *phosphate buffered saline (PBS)* pH 7.0 (PBS; Gibco, Thermo Fisher Scientific, Waltham, MA, USA) for 30 s, sonicated for 30 s (Branson 2510R-MT Ultrasonic Cleaner, Branson, Connecticut, USA), and centrifuged at speed of 13,733×*g* at 4 °C for 20 min. A supernatant was stored at −80 °C until it was ready to be analyzed. An extract was thawed for 15 min and taken 0.1 mL to mixed with 0.1 mL phenolphthalein β-D-glucuronide (Sigma-Aldrich, Merck, St. Louis, MO, USA) at 0.8 mL PBS pH 7.0 for 60 min at 37 °C. The reaction was stopped by adding 2.5 mL of base glycine solution (Merck, Darmstadt, Germany), 1.0 mL of 5% trichloroacetic acid solution (Sigma Aldrich, Merck, St. Louis, MO, USA) and 1.5 mL of distilled water. The color was formed for 10 min and measured by a UV–Vis spectrophotometer at 540 nm (U-2900, Hitachi, Tokyo, Japan). Phenolphthalein release was measured through phenolphthalein curve standard (Sigma-Aldrich, Merck, St. Louis, MO, USA) and protein levels were calculated using the Bradford method with Bovine Serum Albumin standard (Sigma-Aldrich, Merck, St. Louis, MO, USA).

### 2.7 Histological preparations

Histological preparations were made following the procedure of Kiernan [24]. Colonic tissue was cleared with xylol (clearing) (Merck, Darmstadt, Germany) and planted in paraffin (embedding); paraffin tissue is used in staining immunohistochemistry and hematoxylin-eosin for analyzed histopathology.

#### 2.7.1 Staining of hematoxylin-eosin

Tissue fixation was conducted by immersing samples into a formaldehyde solution (Merck, Darmstadt, Germany). Tissue samples that became histopathic were immersed in xylol. The samples were then immersed in a series of alcohol, namely 100%, 90% and 70% ethanol (Merck, Darmstadt, Germany). Next, the samples were soaked in distilled water and soaked in a hematoxylin solution for 5 min (Merck, Darmstadt, Germany). The sample was then placed under running water until the blue color on the slide disappears and the only blue color left was on the colored organ. The next stage included staining the tissue sample with eosin to color the cell cytosol in it (Merck, Darmstadt, Germany).

The sample then entered a multilevel alcohol immersion stage with concentrations of 70%, 95% and absolute alcohol (100%), respectively. Next, the slides were wiped using tissue on parts that were still wet to make sure they were completely dry. Following that, it was dipped into 3 xylol tubes. Finally, the tissue samples were covered with slide cover.

#### 2.7.2 Immunohistochemical staining

This step began with the deparaffinization process by soaking the tissue in the object glass in xylol solution. The tissue was then immersed in a multilevel alcohol solution, starting from high to low concentrations, respectively. The deparaffinization process ended with the immersion of the tissue in distilled water. Next, the object glass was blocked by endogen peroxide (Biogear, Bioventures, Inc, Murfreesboro, Tennesse, USA) and retrieved in the de-clocking chamber. After 45 min, the object glass was cooled and washed in PBS. Following that, it was given an excell block and primary antibodies, namely anti-caspase-3 (ab4051, Abcam, Cambridge, Massachusetts, USA), anti-caspase-8 (ab25901, Abcam, Cambridge, Massachusetts, USA) and anti-Proliferating Cell Nuclear Antigen antibodies ([PC10] ab29, Abcam, Cambridge, Massachusetts, USA) for the night.

After the night, the primary antibody solution was washed and given an excell link (Biogear, Bioventures, Inc, Murfreesboro, Tennesse, USA), goat antirabbit igG HRP-linked antibody as a secondary antibody (Biogear, Bioventures, Inc, Murfreesboro, Tennesse, USA), diaminobenzidine (Biogear, Bioventures, Inc, Murfreesboro, Tennesse, USA), and hematoxylin, respectively. The dehydration process began with the immersion of the tissue in a multilevel pro-analysis ethanol solution. Following that, the tissue was immersed in xylol solution, covered with slide cover and placed under observation.

### 2.8 Data analysis

This study used a completely randomized design, and all data were presented as mean ± SD. On the histopathology of hematoxylin-eosin staining, the tissue damage level was measured on a score from 0 to 4, from no damage to the worst damage, respectively. Histopathological changes observed in tissues based on immunohistochemical staining were grouped according to the un-localized DAB brown color; this was carried out by giving a score based on the level of brown density in the area formed [25]. The scores ranged from 0 (there was no brown area, 0%), 1 (brown was very less concentrated, ±1–25%), 2 (brown was less concentrated, ±26–50%), 3 (deep brown color, ±51–75%), to 4 (very dark brown color, ±76–100%) and were measured using the ImageJ software version 1.39o [26]. Qualitative data were presented descriptively, while quantitative data were analyzed statistically by one-way ANOVA using SPSS version 17 [27]. Moreover, differences in the variance test results were followed by the Duncan test at the level of 5%.

## 3 Results and discussion

### 3.1 Daily diet consumption

The results indicate no significant difference in daily diet consumption between the normal group (C−) and the cancer groups (C+ and BRB) (Table 2). However, daily diet consumption in the BRB group tended to be higher than in the C− and C+ groups. One reason for this was suspected to be that the BRB modified diet contained several flavor components derived from black rice bran, making the palatability of mice in the BRB group higher than in the control group, which was fed cellulose fibre. Zeng et al. [28] reported the prominent flavor in rice bran to be ketones, esters, alcohols, and alkanes, which have a pleasant odor.

**Table 2:** The average daily diet consumption.

Groups	Daily diet consumption (g)
C−	4.09 ± 1.03^a^
C+	3.97 ± 0.52^a^
BRB	4.58 ± 0.45^a^

All data were presented as mean ± SD of eight replications. Different superscripts on the same column showed a significant difference (p < 0.05).

### 3.2 Weight gain

The results indicate significant of weight gain between the control group (C+ dan C− groups) and BRB group (Table 3). The lowest weight gain was found in group C+ (6.09 ± 3.60) indicating the clinical condition of the mice that was the most degraded and more stressed higher than the other groups. One indicator of aches, pains and stress in animals is a decrease in ration intake [29]. Mice in the BRB group experienced clinical changes due to carcinogen induction, but the average daily diet consumption was still higher (13.53 ± 9.63) than the control groups.

**Table 3:** The average increase in body weight.

Groups	Weight gain (%)
C−	9.81 ± 4.97^a^
C+	6.09 ± 3.60^a^
BRB	13.53 ± 9.63^b^

All data were presented as mean ± SD of eight replications. Different superscripts on the same column showed a significant difference (p < 0.05).

### 3.3 Total of LAB

The results suggest that the BRB modified diet was able to increase the total of LAB and SCFAs concentration, while the standard diet was not (Table 4). This might be caused by cellulose fibre in the standard diet being poorly fermented by colonic microflora, and as a result decreasing the growth population of LAB, leading to low-SCFAs and high pH. This theory is supported by the findings of Holscher [30], which indicate that cellulose, an insoluble fibre, was poorly fermented by gut flora than pectin and fructooligosaccharides as a soluble fibre. Chatterjee et al. [31] also report that pectin is known to stimulate the growth of *Lactobacillus acidophilus, Lactobacillus casei* and *Bifidobacterium bifidum,* and that β-glucans could enhance the growth rate of *Lactobacillus plantarum* [32]. These soluble fibres, such as gum, pectin and beta-glucan, have been reportedly contained in rice bran [7]. In our preliminary study, black rice bran contained 6.89% soluble dietary fibre and 16.17% insoluble dietary fibre. This reinforces our theory of why mice fed the BRB diet showed a greater BAL population than those supplied with the standard diet.

**Table 4:** The total LAB (Log CFU/g) in mice faeces and cecum.

Observation Conditions	Groups		
	C−	C+	BRB
A	8.11 ± 0.15^a^	8.25 ± 0.14^a^	8.41 ± 0.00^a^
B	8.13 ± 0.04^a^	8.06 ± 0.04^a^	8.46 ± 0.02^b^
C	8.02 ± 0.09^b^	7.27 ± 0.21^a^	8.13 ± 0.01^b^
D	7.99 ± 0.03^b^	6.97 ± 0.56^a^	8.24 ± 0.09^b^
E	7.33 ± 0.05^a^	7.10 ± 0.01^a^	8.26 ± 0.18^b^

All data were presented as mean ± SD of eight replications. Different superscripts on the same line showed a significant difference (p < 0.05) A: faeces with all mice given standard diet, B: faeces with the control group given standard diet and BRB given black rice bran modified diet, C: 8 weeks after AOM induction, D: 16 weeks after AOM induction, E: Cecum contents were taken after euthanasia.

The growth of bacteria such as *Clostridium perfringens*, *Clostridium difficile*, and Bactreriodes can be inhibited by a bioactive compound that is abundant in black rice bran [33]. The mechanism of bioactive compounds in inhibiting pathogens is to bind to bacterial cell membranes and interfere with the activity of the cell membrane[[34]]. Another hypothesis by Calabrese et al.[[35]] namely the formation of iron bioactive-ion complexes which causes iron deficiency in the colon. This causes a population of bacteria will change especially aerobic bacteria. Aerobic bacteria need iron to carry out their functions such as reducing ribonucleotide precursors from DNA and forming a heme group.

### 3.4 pH value

As SCFAs were produced in the mice’s colon, the colonic pH decreased. The pH value of the faeces in the C+ and C- groups was not significantly different (Table 5). It was due to the fiber in the diet being cellulose. Cellulose is poorly fermented in the gut, so the pH decrease is not too significant [28]. Black rice bran contains soluble dietary fiber, which microbes can ferment in the colon to produce SCFAs, which can lower colonic pH [36]. Decline colonic pH causes inactivation of the 7α-dehydroxylase enzyme, which can form secondary bile acids. Secondary bile acid is one promoter of colon cancer [4].

**Table 5:** The pH value in mice faeces and cecum.

Observation Conditions	Groups		
	C−	C+	BRB
A	7.95 ± 0.09^a^	8.02 ± 0.10^a^	8.01 ± 0.07^a^
B	7.97 ± 0.01^a^	7.85 ± 0.19^a^	7.77 ± 0.08^a^
C	7.70 ± 0.11^a^	7.85 ± 0.01^a^	7.39 ± 0.02^b^
D	8.07 ± 0.14^a^	8.17 ± 0.06^a^	7.38 ± 0.06^b^
E	7.56 ± 0.13^a^	7.59 ± 0.01^a^	7.37 ± 0.03^a^

All data were presented as mean ± SD of eight replications. Different superscripts on the same line showed a significant difference (p < 0.05) A: faeces with all mice given standard diet, B: faeces with the control group given standard diet and BRB given black rice bran modified diet, C: 8 weeks after AOM induction, D: 16 weeks after AOM induction, E: Cecum contents were taken after euthanasia.

The pH value of the cecum contents was not significantly different for all treatment groups. However, the pH value of the cecum contents in all groups was lower than the pH of faeces at 16 weeks post-induction AOM. Due to the fermentation of soluble dietary fiber in rodents occurring in the cecum, the number of microbes in the cecum is more compared to faeces. Therefore, the cecum contents' pH value is lower than faeces [29].

### 3.5 SCFAs concentration

The results suggest that the BRB-modified diet increased the concentration of SCFAs (Table 6). It might be caused by cellulose fibre in the standard diet being poorly fermented by colonic microflora. As a result, it decreased SCFAs concentration, and black rice bran, which is high in dietary fiber, is fermented by colonic microflora as prebiotics.

**Table 6:** The concentration of SCFAs (mmol) in mice faeces and cecum.

Observation Condition	Types	Groups		
		C−	C+	BRB
A	Acetic	227.68 ± 5.56^a^	226.85 ± 2.37^a^	221.73 ± 1.23^a^
	Propionic	6.75 ± 0.05^b^	14.46 ± 0.16^a^	7.28 ± 0.01^c^
	Butyric	11.25 ± 0.96^b^	16.68 ± 0.26^a^	10.06 ± 1.42^b^
	SCFA total	260.14 ± 4.39^b^	274.59 ± 2.89^a^	249.13 ± 0.49^c^
B	Acetic	169.48 ± 6.28^a^	166.40 ± 2.39^a^	191.86 ± 0.86^b^
	Propionic	12.00 ± 0.20^a^	9.71 ± 1.52^a^	26.00 ± 0.01^b^
	Butyric	7.35 ± 2.22^a^	7.11 ± 1.36^a^	8.43 ± 0.81^a^
	SCFA total	194.37 ± 9.26^a^	188.36 ± 5.99^a^	238.54 ± 0.45^b^
C	Acetic	111.83 ± 5.44^a^	120.06 ± 3.14^a^	162.21 ± 5.91^b^
	Propionic	3.21 ± 0.50^a^	3.57 ± 0.00^a^	13.92 ± 4.54^b^
	Butyric	7.68 ± 1.77^a^	5.89 ± 1.77^a^	28.81 ± 4.39^b^
	SCFA total	128.15 ± 7.31^a^	136.95 ± 6.53^a^	222.08 ± 0.60^b^
D	Acetic	54.33 ± 2.79^a^	49.36 ± 1.09^a^	98.01 ± 4.06^b^
	Propionic	13.69 ± 0.78^a^	19.61 ± 0.24^a^	19.82 ± 3.49^a^
	Butyric	12.32 ± 1.87^a^	6.21 ± 1.71^a^	22.82 ± 2.88^b^
	SCFA total	88.21 ± 0.18^a^	89.29 ± 4.30^a^	157.27 ± 13.32^b^
E	Acetic	116.68 ± 4.19^b^	47.68 ± 5.93^a^	123.88 ± 3.19^b^
	Propionic	13.39 ± 0.18^a^	9.76 ± 3.52^a^	18.86 ± 3.32^a^
	Butyric	14.62 ± 4.01^ab^	6.69 ± 2.03^a^	15.43 ± 0.28^b^
	SCFA total	149.03 ± 15.00^b^	68.41 ± 12.85^a^	166.65 ± 5.74^b^

All data were presented as mean ± SD of eight replications. Different superscripts on the same line showed a significant difference (p < 0.05) A: faeces with all mice given standard diet, B: faeces with the control group given standard diet and BRB given black rice bran modified diet, C: 8 weeks after AOM induction, D: 16 weeks after AOM induction, E: Cecum contents were taken after euthanasia.

This LAB fermentation results in SCFAs products, such as acetate, propionate and butyrate, which are protective against colon cancer [4]. In this research, the predominant SCFAs product found in both the faeces and cecum was acetic acid (Table 6). According to den Besten et al. [37], acetic acid is dominantly found in the faeces and colon, followed by propionic and butyric acid.

Interestingly, in faeces of the BRB group, the concentration of butyric acid was found higher than propionic acid in weeks 8–16 (C-D), but a decline occurred during weeks 17 (cecum) (E). Butyric acid has been reported to possess protective factors against colon cancer, such as upregulate expression of p21 tumor suppressor gene [38] and upregulate of Wnt activity [39] meaning that it could inhibit the proliferation and apoptosis induction of cancer.

Acetic and propionic acid are also known as inhibitors of colorectal cancer development; for example, acetate has the ability to induce lysosomal membrane permeabilization and release cathepsins, thereby inducing the death of cancer cells [40]. Propionate can inhibit cancer cells' growth and differentiation through the hyperacetylation of histone protein and apoptosis [41]. However, butyric acid has been reported as the stronger inhibitor on CRC cells compared to acetic and propionic acid [[41], [42]]. The high levels of butyric, propionic and acetic acid in faeces and cecum in the BRB mice group indicates that the dietary fiber contained in rice bran might be more effective to inhibit colon cancer than cellulose fiber.

In the faeces of the BRB group, the concentration of total SCFAs at week 16 post-induction of AOM was a significant decrease compared to pre-induction conditions when mice were given a standard diet. However, the total of butyric in the BRB group increased significantly compared to the pre-induction condition when mice were given a standard diet. In contrast, the concentration of butyric in the C+ group decreased significantly compared to pre-induction conditions when mice were given a standard diet. It shows that fermentation of soluble dietary fiber from black rice bran can produce higher butyric acid than mice fed the standard diet. In addition, it also shows that butyric is more protective against the prevention of colon cancer development than acetic and propionic [43].

Cummings and MacFarlane [44] stated that the number of SCFAs resulting from soluble dietary fiber fermentation is 62% acetic, 25% propionic, and 16% butyric. Diet composition and microbial total in the colon is the main factor that can increase SCFAs. These SCFAs are ready to be absorbed by the colon and used as an energy source for the colon. Butyric acts as the primary energy source of the colon and is most important in preventing and treating colonic mucosal diseases such as colon cancer. Butyric acid can also increase cell differentiation and tumor cell apoptosis [45]. Butyric can also increase cell apoptosis through decreasing levels of the anti-apoptotic gene, namely Bcl-2, increasing the Bax gene, which is a proapoptotic gene, and induces apoptosis via caspase-3 [46].

### 3.6 β-Glucuronidase activity

The results show a higher increase of specific and total activity of β-glucuronidase in the wall of mice cecum in the C+ group compared to the normal/healthy group (Table 7). This suggests that β-glucuronidase accumulates higher in the colon bacteria of mice induced by AOM than in the normal mice bacteria (C−). Although the specific activity of β-glucuronidase in BRB was lower than in C+ and C− groups, it shows that fiber from black rice bran can reduce the activity of β-glucuronidase. The SCFAs are produced due to soluble dietary fiber fermentation in black rice bran, causing a decrease in colonic pH conditions. Colon conditions that tend to be acidic are not favored by *Escherichia coli* and Clostridium, which produce β-enzyme glucuronidase [46]. Other intestinal bacteria which have been reported to produce β-glucuronidase enzymes are Bacteroides and some anaerobes of obligate [47]. Kim and Jin [48] report that β-glucuronidase activity in the sonicated fecal of colon cancer patients was found 12.1 times higher than that of healthy persons, meaning that bacterial β-glucuronidase enzyme is associated with occurrence of this cancer.

**Table 7:** The average total activity and specific activity of the β-glucuronidase enzyme in the wall of mice cecum at 17 weeks after AOM induction.

Groups	Specific Activity (nmol phenolphthalein/mg protein cecum/min)	Total activity (nmol phenolphthalein/cecum/min)
C−	48.72 ± 12.04^a^	173.76 ± 31.63^b^
C+	60.59 ± 19.00^a^	360.29 ± 74.19^a^
BRB	49.67 ± 25.40^a^	222.85 ± 17.05^b^

All data were presented as mean ± SD of eight replications. Different superscripts on the same column showed a significant difference (p < 0.05).

On the contrary, specific activity and total activity of β-glucuronidase in the BRB group was lower than in the C+ group and almost the same as in the normal group (C−) (not significant). This could be because an of increase in the growth population of LAB in both faeces and cecum of BRB mice after they were fed a BRB diet, thus inhibiting gut bacterial *β-glucuronidase*. Sreekumar and Hosono [47][46] indicate that *Lactobacillus acidophilus* can inhibit gut flora such as *coliforms, E. coli* and anaerobes as well as β-glucuronidase enzymes. Furthermore, a lower colonic pH in the BRB group (Table 7) obstructed the growth of intestinal pathogenic bacterial and inhibited the β-glucuronidase activity [49].

### 3.7 Histopathology of colon tissue

**Fig. 2:**
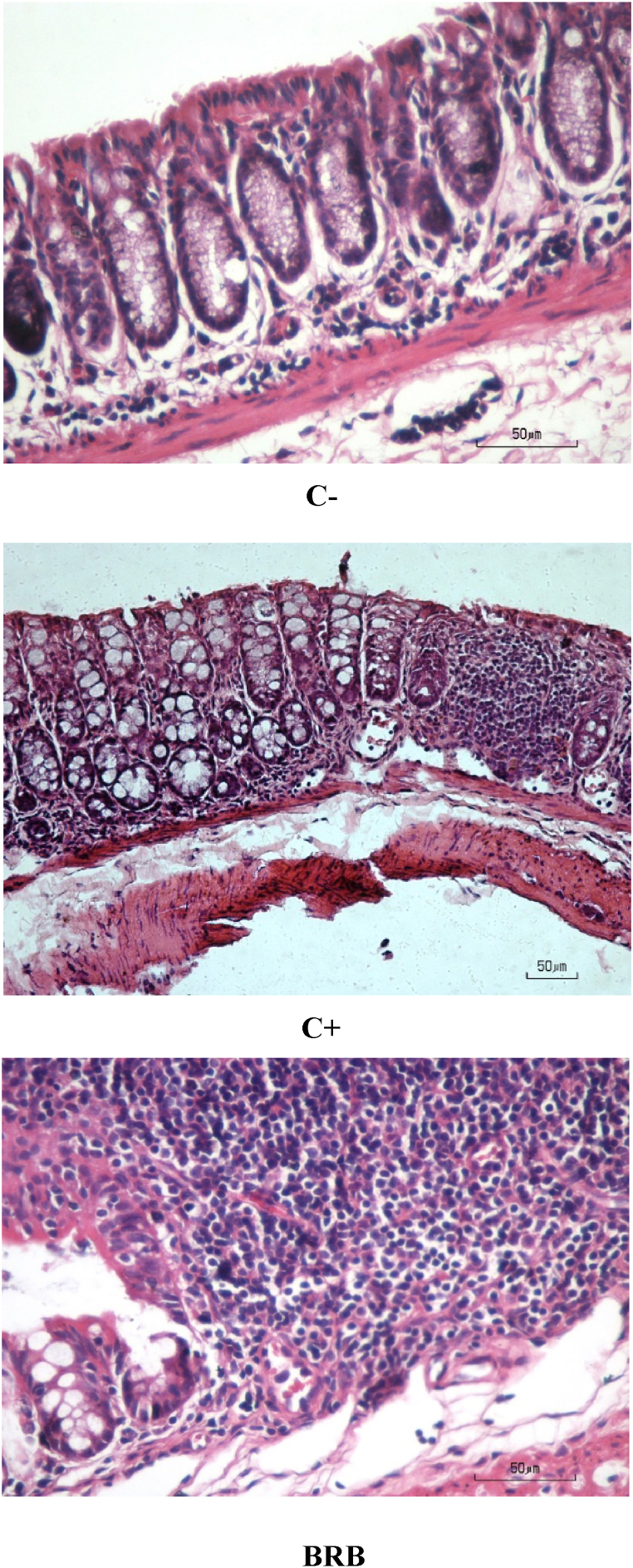
Photomicrograph tissue of mice colon with hematoxylin-eosin staining. C- (normal colon), C+ (carcinogen-induced colon), BRB (carcinogen-induced colon fed by BRB modified diet). Magnification of 100×.

The results of the histopathological evaluation of mucosal tissue of the C− group with the cancer groups (C+ and BRB) can be seen in Fig. 2, which depicts the morphological differences between the C- and cancer groups. The C− group exhibited a normal/healthy colon mucosa of mice, which could be seen from the uniform tissue cell and nucleus size and the unchanged space between cells. In contrast, the C+ group appeared to have lost their cryptic form, indicating that their cells had been damaged and suggesting the presence of severe intestinal inflammation (colitis) in the cell mucosa. In addition, there was an acute inflammatory cell infiltration of the mucosal, submucosal and mucosal muscularis though the surface epithelium. The colon tissue of the C+ group was in the dysplasia stage (score 3).

The administration of black rice bran fibre to the BRB mice group showed the presence of mild inflammation and the occurrence of inflammatory cells. Colonic tissue that was inflamed was evident from the shortened of 2/3 lost crypts and the infiltration of a number of inflammatory cells which occurred at the hyperplasia stage (score 2). However, dysplasia was also found in some mice of the BRB group. Lesion carcinoma was not found in the BRB group, similar to in the C+ group. Based on the histopathological appearance, findings of the BRB group show that the rate of their tumor development was inhibited along with the feeding of black rice bran as a fibre substitute. This can also be seen in Table 8, which shows decrease colitis markers in the BRB group (2.67 ± 0.52) compared to the C+ group (2.83 ± 0.41), although the C+ and BRB groups were not significantly different.

**Table 8:** Testing of colitis markers in mice colon with hematoxylin-eosin staining.

Groups	Colitis Marker Score Average
C−	0,00 ± 0,00^a^
C+	2,83 ± 0,41^b^
BRB	2,67 ± 0,52^b^

All data were presented as mean ± SD eight replications. Different superscripts on the same column showed a significant difference (p < 0.05).

Colonic mucosal damage in the C+ group indicates that fibre cellulose was unable to suppress the occurrence of inflammation and cancer formation due to AOM/DSS carcinogens. This could be due to the high β-glucuronidase activity in the wall of mice cecum in the C+ group (Table 7). The carcinogenic AOM compounds injected into the C+ mice group enabled the occurrence of hydroxylation by microsomal monooxygenase systems in the livers of the mice, conjugated with glucuronic acid until carried to the colon via bile. The presence of β-glucuronidase in the colon caused hydrolyzed conjugate, thus MAM was free and accumulated more in the colonic mucosa. Consequently, the production of alkylating methyl carbonium ion which reacts with molecular and cellular targets which were the initial of colon carcinogenesis [50].

β-Glucosidase activity was a key factor in measuring the accuracy of colon carcinogenesis, because it was needed to metabolize AOM [48]. This also reinforces the suspicion of an inhibition of the rate of colon cancer development in the BRB group. A lower β-glucuronidase activity in the BRB group enables partial conjugation with glucuronic acid to be secreted. Takada et al. [50] concluded that the inhibition of β-glucuronidase lead carcinogens conjugated with glucuronic acid is secreted through faeces, thus colon carcinogenesis by AOM can be prevented.

### 3.8 Surface markers proliferating cell nuclear antigen (PCNA), caspase-3 and caspase-8 with immunohistochemical staining

**Fig. 3:**
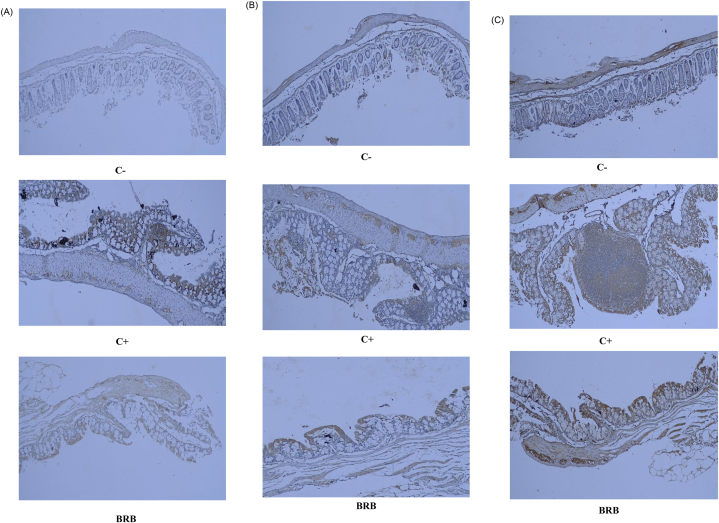
Photomicrograph of mice colon tissue with histopathology of immunohistochemistry. a) anti-PCNA antibodies, b) anti-caspase antibodies 3, c) anti-caspase antibodies 8. C- (normal colon), C+ (carcinogen-induced colon), BRB (carcinogen-induced colon fed by BRB modified diet). Magnification 10×.

As can be seen in Table 9, the C+ group expressed significantly higher PCNA (12.00 ± 0.00) than the other groups. Interestingly, the PCNA marker score in the BRB group was not significant with the C− normal group, these were 1.50 ± 0.00 and 1.00 ± 0.00, respectively, meaning that mice fed the rice bran diet had the potential to suppress cell proliferation during cancer development. The findings concerning the photomicrograph tissue of colon show that the C+ group had a more intense brown color in cell nucleus compared to the BRB and C- groups (Fig. 3a). The brown intensity in the cell nucleus was formed because of the expression of PCNA which indicated the proliferation of DNA cells.

**Table 9:** Testing of PCNA, caspase-8 and 3 markers in mice colon with immunohistochemical staining using anti- PCNA and anti-caspase 8 and 3 antibodies.

Groups	PCNA Marker Score	Caspase-3 Marker Score	Caspase-8 Marker Score
C-	1.00 ± 0.00^a^	1.00 ± 0.00^a^	1.00 ± 0.00^a^
C+	12.00 ± 0.00^b^	3.83 ± 0.23^b^	6.00 ± 0.00^b^
BRB	1.50 ± 0.53^a^	5.67 ± 0.81^c^	12.46 ± 0.9^c^

All data were presented as mean ± SD eight replications. Different superscripts on the same column showed a significant difference (p < 0.05).

The mechanisms that allow PCNA inhibition are SCFAs products such as butyric and acetic acid. Both were reported to be able to stimulate the expression of p21 protein through independent p53 growth control pathways [51]. This active p21 gene is able to bind to the active site on PCNA, and, consequently, the process of DNA replication and cell cycle progression is stopped [52]. The high levels of butyric and acetic acid in the cecum of the BRB group mice at week 17 (Table 6) confirm the finding of the alleged low level of PCNA activity.

On Fig. 3b and c, the brown color in the cell nucleus on the BRB group is more intense compared to the C+ group. Based on the calculation of the intensity values presented in Table 9, the caspase-3 and -8 expressions in the BRB group were 5.67 ± 0.81, 12.46 ± 0.9 respectively; both caspase expressions are significantly higher than those of the C+ group. This shows that the BRB group of cancer cells undergo much apoptosis. Apoptosis refers to programmed tumor cell death, and its function is very important to maintain cell homeostasis [8].

Butyric acid has been known to induce apoptosis through the mitochondrial pathway; butyric acid alters the permeability of the mitochondrial membrane and forms ion channels, and this membrane change causes the apoptotic molecules of mitochondria to emerge, namely cytochrome c and apoptosis, inducing factor (AIF) [45]. Another study reports that the cycloartenyl ferulate in rice bran can increase caspase-3 and caspase-8 activity in SW620 and SW480 cells [53]. Therefore, we suspect that the mice fed the rice bran modified diet had better colon cancer inhibition because black rice bran contains various bioactive compounds and potential dietary fibre.

## 4 Conclusions

Provision of black rice bran to BALB/c mice diet induced by colon carcinogen was able to inhibit colorectal cancer development, as discovered from the increase of the total of LAB after AOM with statistical values not different at the group of normal mice at 16 weeks. Then the rice bran diet was effective in reducing pH and β-glucuronidase activity compared to cancer mice that were given a standard AIN-93M diet with cellulose as fiber. LAB was found to have the ability to ferment soluble dietary fibre from BRB and produce the highest SCFAs product, which is protective against colorectal cancer. Based on histopathological observations of colon tissue, tumor development was inhibited along with BRB administration, which was also characterized by decreased cell proliferation, and induction of the apoptotic pathways (caspase-3 and caspase-8) in the colon of BALB/c mice.

## Author contribution statement

Slamet Budijanto: Conceived and designed the experiments; Analyzed and interpreted the data; Contributed reagents, materials, analysis tools or data.

Winda Nurtiana, Amirotul Muniroh, Yeni Kurniati: Performed the experiments; Analyzed and interpreted the data; Wrote the paper.

Lilis Nuraida, Bambang Pontjo Priosoeryanto, Fitriya Nur Annisa Dewi: Conceived and designed the experiments; Analyzed and interpreted the data.

Ardiansyah, Nancy Dewi Yuliana, Safrida, Hitoshi Shirakawa: Analyzed and interpreted the data; Wrote the paper.

## Data availability statement

Data included in article/supp. material/referenced in article.

## Declaration of competing interest

Authors declare no conflict of interest in this research. All procedures involving the care and use of animals in this research were performed in compliance to national and institutional standards on animal welfare and ethics, following the approval from PT. Bimana Indomedical Institutional Animal Care and Use Committee (approval number R.02-17-IR).
